# Genome Sequence of an *Alkaliphilus* Species Isolated from Historically Contaminated Sediments of the Gulf of Naples (Mediterranean Sea)

**DOI:** 10.1128/MRA.00060-21

**Published:** 2021-03-18

**Authors:** Filippo Dell’Anno, Leonardo Joaquim van Zyl, Marla Trindade, Christophe Brunet, Antonio Dell’Anno, Adrianna Ianora, Clementina Sansone

**Affiliations:** a Stazione Zoologica Anton Dohrn, Istituto Nationale di Biologia, Ecologia, e Biotecnologie Marine, Naples, Italy; b Institute for Microbial Biotechnology and Metagenomics, Department of Biotechnology, University of the Western Cape, Cape Town, South Africa; c Department of Life and Environmental Science, Università Politecnica delle Marche, Ancona, Italy; Georgia Institute of Technology

## Abstract

Here, we report the draft genome sequence of a metagenome-assembled genome (MAG) of a new *Alkaliphilus* bacterium, NP8, of the *Clostridiaceae* family. This bacterium was isolated from polluted sediment collected from an abandoned industrial site located in the Gulf of Naples (Mediterranean Sea) as part of a microbial consortium.

## ANNOUNCEMENT

**FIG 1:**
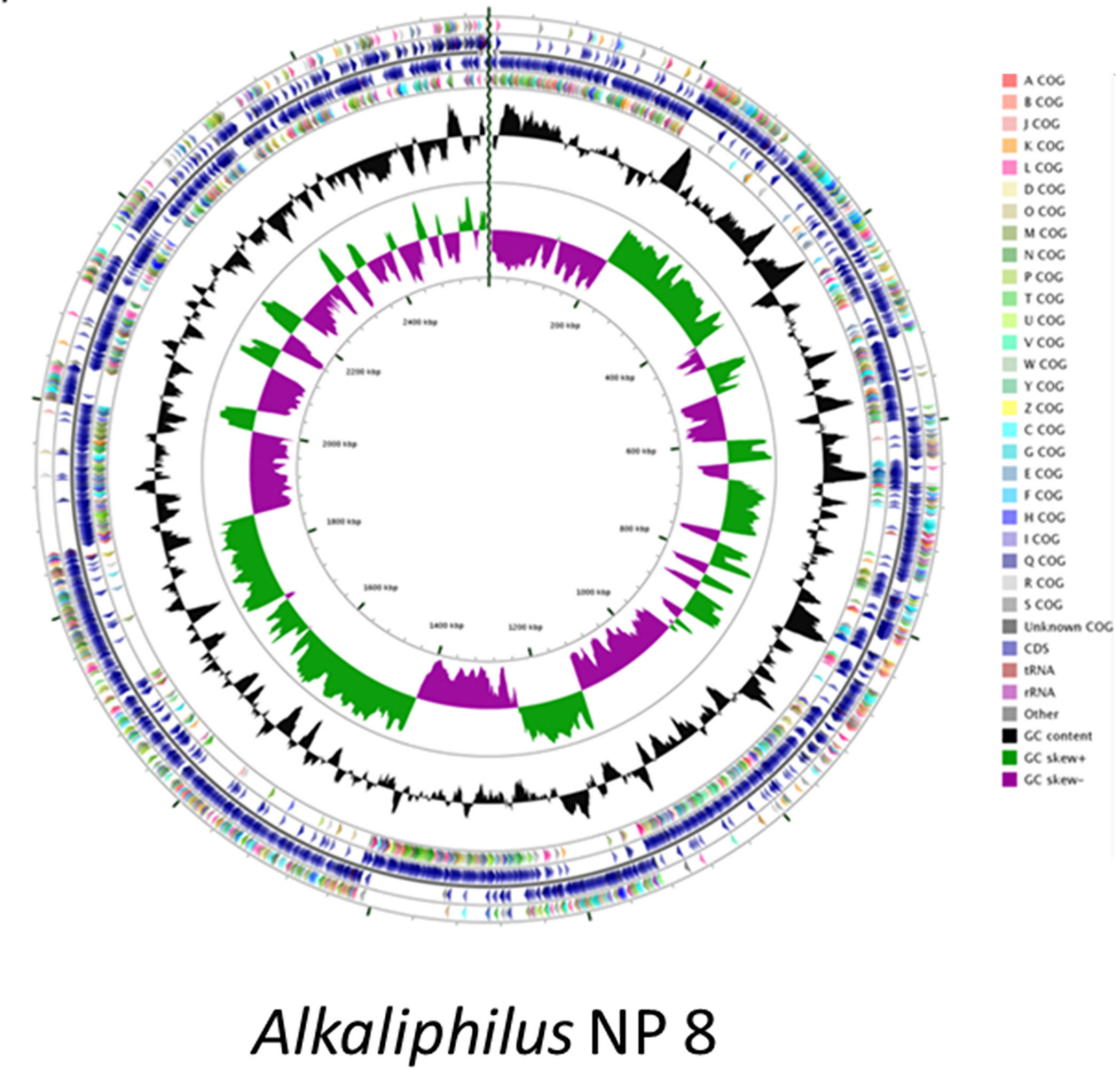
Circular representation of the *Alkaliphilus* bacterium NP8 genome using CGView Server^Beta^ (http://cgview.ca). The different rings represent (from outer to inner) predicted protein-coding sequences (CDS) on the forward (outer wheel) and reverse (inner wheel) strands (rings 2 and 3) colored according to the assigned Cluster of Orthologous Groups (COG) classes (rings 1 and 4), GC content (ring 5), GC skew (ring 6), and genomic position (ring 7). The key indicates the COG colors for the functional groups (A, RNA processing and modification; B, chromatin structure and dynamics; J, translation, ribosomal structure, and biogenesis; K, transcription; L, replication, recombination, and repair; D, cell cycle control, cell division, and chromosome partitioning; O, posttranslational modification, protein turnover, and chaperones; M, cell wall/membrane/envelope biogenesis; N, cell motility; P, inorganic ion transport and metabolism; T, signal transduction mechanisms; U, intracellular trafficking, secretion, and vesicular transport; V, defense mechanisms; W, extracellular structures; Y, nuclear structure; Z, cytoskeleton; C, energy production and conversion; G, carbohydrate transport and metabolism; E, amino acid transport and metabolism; F, nucleotide transport and metabolism; H, coenzyme transport and metabolism; I, lipid transport and metabolism; Q, secondary metabolite biosynthesis, transport, and catabolism; R, general function prediction only; S, function unknown).

The ability of *Alkaliphilus* species to deal with harsh environmental conditions was described previously (1), e.g., an Alkaliphilus transvaalensis strain isolated from an ultradeep (3.2 km below the surface) gold mine. The metal-reducing capability reported for *Alkaliphilus* members (2, 3) likely plays a pivotal role in survival under extreme conditions. We report the metagenome-assembled genome (MAG) of a novel *Alkaliphilus* bacterium cultured as part of a microbial consortium from homogenized superficial marine sediments (0 to 20 cm) sampled through a Van Veen grab sampler in the Gulf of Naples (40°48′29.0″N, 14°09′54.7″E), which is highly contaminated by heavy metals and hydrocarbons (4). For sequencing, DNA was extracted from an enriched mixed microbial culture from marine sediment with the DNeasy blood and tissue kit (Qiagen) according to the manufacturer’s instructions. Sequencing library preparation was performed using the Nextera DNA Flex kit (Illumina, Hayward, CA, USA) with 1 ng input DNA according to the manufacturer's instructions. The resulting libraries were sequenced on an Illumina MiSeq platform at the University of the Western Cape (Cape Town, South Africa) sequencing facility using a MiSeq reagent kit v2 (500 cycles) with a 10% phiX v3 spike generating 2 × 250-bp reads. Metagenome assembly was performed using CLC Genomics Workbench v7.5.1. The raw reads were trimmed and demultiplexed, and ≤500-bp contigs were removed from the final assembly. Binning of metagenomic contigs was performed using MyCC (https://sourceforge.net/projects/sb2nhri/files/MyCC) (5), while the completeness and contamination of the MAG, as well as genome quality, were determined with CheckM v1.0.18 using the lineage-specific workflow and default parameters (6). Gene prediction and annotation were performed using the Rapid Annotation using Subsystem Technology (RAST) pipeline (http://rast.nmpdr.org) (7) and the MicroScope pipeline (https://mage.genoscope.cns.fr/microscope/home/index.php) (8). The draft genome of *Alkaliphilus* NP8 is composed of 81 contigs totaling 2,673,585 bp, with a GC content of 29.1%, containing 2,661 putative genes with an average length of 822 bp (Fig. 1). CheckM analysis showed completeness of 98.6% and contamination of 0.6% (Table 1).

**TABLE 1:** General features of the genome of *Alkaliphilus* NP8

Parameter	Finding
CheckM results	
Completeness (%)	98.6
Contamination (%)	0.6
Size (bp)	2,673,585
GC content (%)	29.1
*N*_50_ (bp)	65,217
*L*_50_	12
No. of contigs (with protein-encoding genes)	81
No. of subsystems	336
No. of coding sequences	2,671
No. with function assigned	1,827
No. hypothetical	844
No. of RNAs	36

The organism belongs to the placeholder genus *Alkaliphilus* B. Taxonomy was assigned through whole-genome assessment against the Genome Taxonomy Database v1.1.0 (https://gtdb.ecogenomic.org) as provided in KBase (https://kbase.us/applist/apps/kb_gtdbtk/run_kb_gtdbtk/release?gclid=CjwKCAiAyc2BBhAaEiwA44-wW7HAIOI9WWahrkAV0qleUu92NZCGEu34cIa4XVbD3Vh0-xGBBH35NRoCOEUQAvD_BwE). To further explore the ability of *Alkaliphilus* NP8 to survive in polluted marine environments, we analyzed the gene functional categories provided by the annotation systems. The effective functionality of the observed genes has yet to be determined. In detail, two genes are involved in resistance to fluoroquinolones, while 18 genes are related to heavy metal detoxification, such as copper homeostasis and tolerance, cobalt, zinc, and cadmium resistance, mercuric reductase, the mercuric resistance operon, and multidrug resistance efflux pumps. We report 22 genes coding for superoxide dismutase and glutathione-related pathways, whose antioxidant and detoxification functions have already been described (9). The presence of genes involved in benzoate degradation, chloroalkane and chloroalkene degradation, naphthalene degradation, aminobenzoate degradation, and quinate degradation suggests the ability of *Alkaliphilus* NP8 to deal with hydrocarbon contamination.

### Data availability.

The draft genome sequence of *Alkaliphilus* NP8 was deposited under accession number JADWMM000000000.1 and BioProject number PRJNA669418. Whole-genome sequencing and Sequence Read Archive (SRA) records are available under accession numbers JADWMM01 and SRR13496755, respectively.
